# High Levels of Sediment Contamination Have Little Influence on Estuarine Beach Fish Communities

**DOI:** 10.1371/journal.pone.0026353

**Published:** 2011-10-19

**Authors:** Andrew C. McKinley, Katherine A. Dafforn, Matthew D. Taylor, Emma L. Johnston

**Affiliations:** Evolution and Ecology Research Centre, School of Biological, Earth and Environmental Sciences, University of New South Wales, Sydney, New South Wales, Australia; University of Hamburg, Germany

## Abstract

While contaminants are predicted to have measurable impacts on fish assemblages, studies have rarely assessed this potential in the context of natural variability in physico-chemical conditions within and between estuaries. We investigated links between the distribution of sediment contamination (metals and PAHs), physico-chemical variables (pH, salinity, temperature, turbidity) and beach fish assemblages in estuarine environments. Fish communities were sampled using a beach seine within the inner and outer zones of six estuaries that were either heavily modified or relatively unmodified by urbanization and industrial activity. All sampling was replicated over two years with two periods sampled each year. Shannon diversity, biomass and abundance were all significantly higher in the inner zone of estuaries while fish were larger on average in the outer zone. Strong differences in community composition were also detected between the inner and outer zones. Few differences were detected between fish assemblages in heavily modified versus relatively unmodified estuaries despite high concentrations of sediment contaminants in the inner zones of modified estuaries that exceeded recognized sediment quality guidelines. Trends in species distributions, community composition, abundance, Shannon diversity, and average fish weight were strongly correlated to physico-chemical variables and showed a weaker relationship to sediment metal contamination. Sediment PAH concentrations were not significantly related to the fish assemblage. These findings suggest that variation in some physico-chemical factors (salinity, temperature, pH) or variables that co-vary with these factors (e.g., wave activity or grain size) have a much greater influence on this fish assemblage than anthropogenic stressors such as contamination.

## Introduction

A variety of anthropogenic activities contribute to widespread modification of estuarine environments, and estuaries are among the most highly impacted of all marine ecosystems [1]. Contamination is a major form of anthropogenic impact in estuarine systems, acting as a stressor which influences the composition and health of ecological communities. Estuaries are generally believed to contain the highest levels of contamination of any marine environment due to their proximity to human settlements and their position directly downstream of agricultural and industrial activities [2]. Many of these complex estuarine habitats provide a ‘nursery’ function for ecologically and economically important species of fish [3]. It has been demonstrated that contaminants in these systems can have substantial impacts on larval fish [4], and that they generally reduce the richness and evenness of marine invertebrate communities [5]. Despite this, few studies have identified impacts of contamination on post-settlement fish or within the context of natural variability in physico-chemical conditions [6]. As a result, the relative importance of contaminant impacts on fish assemblages compared to natural hydrographic variability is poorly understood. Identifying stressors and monitoring ecological impacts in post-settlement estuarine fish communities is critical to managing and conserving native biodiversity in these systems.

Toxicants such as metals and organic Polycyclic Aromatic Hydrocarbons (PAHs) are found in fish at various stages of their life cycle, often at levels that may potentially reduce growth or survivorship [7]. Toxic substances may have adverse effects on fish by interfering with reproductive processes and by causing developmental problems [8]. However, impacts of contaminants on post-settlement fish assemblages have been shown to be highly variable; many studies have reported either localized impacts or no effect of contaminants on marine fish assemblages, and negative impacts at large scales are rarely described [6]. Different types and concentrations of contaminants may have either toxic or enriching effects on fish assemblages, however the effects differ between different guilds of fish [9]. In many cases, contaminants with enrichment properties (such as nutrients or sewage) have a largely positive effect on the abundance and diversity of post-settlement fish [6].

While toxicants are thought to have significant impacts on wild fish communities, natural variation in physico-chemical conditions such as changes in turbidity, salinity, temperature and pH have consistently been shown to have a large influence on the composition and species richness of fish assemblages [10]. Spatial variation in physico-chemical factors can manifest as gradients within estuarine systems, influencing the distribution of fish species along the length of an estuary [11]. Similarly, seasonal and temporal variability in physico-chemical factors can influence fish communities [12]. Estuary geomorphology and physical structure will also affect estuarine ecology through variation in entrance conditions (e.g. permanently or intermittently open estuaries), the relative size of the fluvial and tidal deltas, and the evolutionary maturity (stage of sediment filling) of the estuarine system [13]. The way in which these factors influence the fish assemblage is increasingly juxtaposed against the effects of anthropogenic modification to estuaries. As such, identifying the major drivers of fish distribution is likely to be more complicated in modified habitats.

Estuarine beaches are a dynamic environment representing a juncture between terrestrial and marine systems. These environments are heavily influenced by both wave action and tidal forces [14]. Due to their shallow nature and position at the shoreline, they are also likely to be relatively heavily impacted by anthropogenic developments situated onshore or within estuarine waters. Beach environments may be directly influenced by run-off from urban environments, shoreline alteration, changes to terrestrial detritus patterns, beach fishing, changes to sediment quality, and physical disturbance from recreational activities [15], [16]. Fish living in these habitats represent a diverse community which is primarily small bodied species or juveniles, feeding on a diverse array of food items including terrestrial and marine detritus, plankton and sediment dwelling invertebrates, marine vegetation, and other fish [17]. As such, estuarine beaches represent a potentially important environment for the study of anthropogenic impacts on fish. While beach fish may be responsive to anthropogenic modification for the reasons discussed, it should be noted that fish which live in sensitive biogenic habitats such as coral reefs and seagrass beds maybe more sensitive to modification and contamination than those in bare habitats, as the biogenic habitat itself may be degraded by these stressors [18], [19].

We explore the impacts of large-scale anthropogenic disturbance on estuarine beach fish communities across heavily modified and relatively unmodified estuaries in New South Wales, Australia. Specifically, we investigate whether high levels of modification and sediment contamination in the estuarine environment influence the composition, abundance, and Shannon diversity of the post-settlement fish assemblage. We assess these impacts within the context of environmental variability both within and between estuaries in order to understand the scale of anthropogenic impacts relative to variation in environmental conditions [20]. While this would ideally be assessed using a Before After Control Impact (BACI) sampling design, baseline data was not available for the study estuaries. As such, we utilize a spatial comparison of heavily modified vs. relatively unmodified estuaries to test our hypotheses [21].

## Methods

### Study Sites

**Figure 1:**
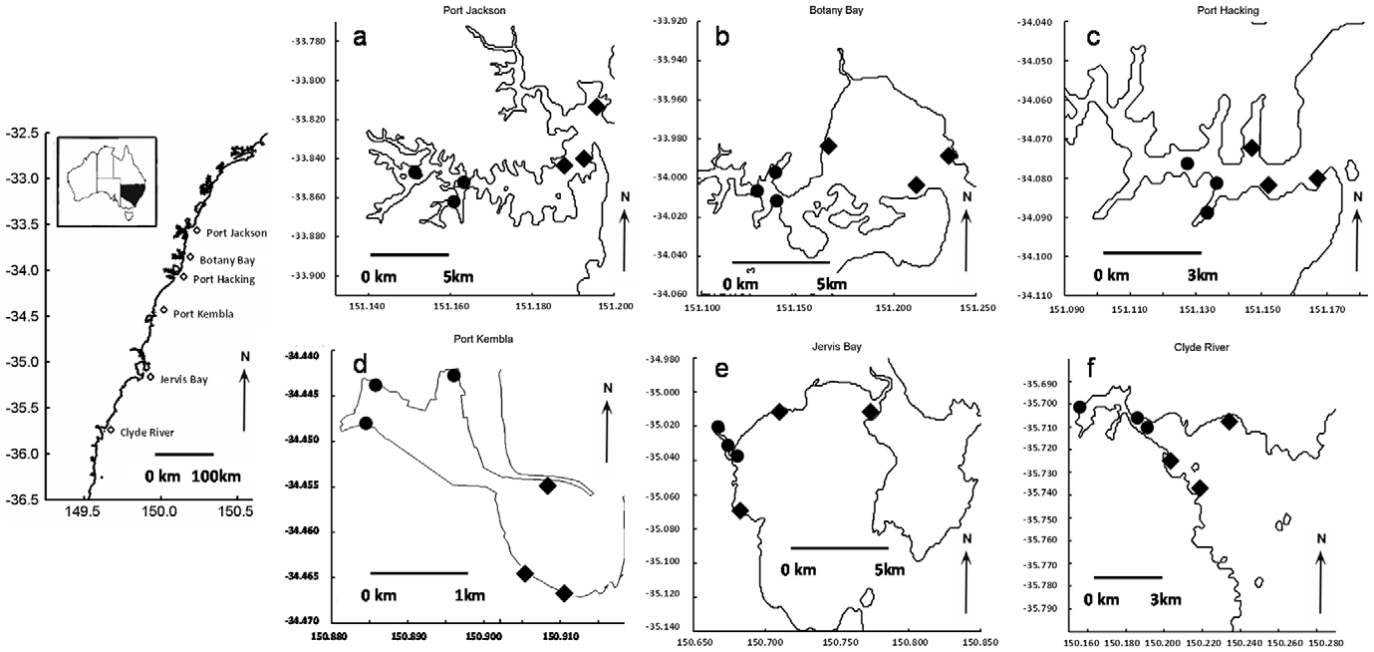
Location of study sites in the six focal estuaries: a) Port Jackson (heavily modified), b) Botany Bay (heavily modified), c) Port Hacking (relatively unmodified), d) Port Kembla (heavily modified), e) Jervis Bay (relatively unmodified), and f) Clyde River (relatively unmodified). Filled diamonds (♦) indicates outer zone sites. Filled circles (•) indicates inner zone sites.

Fish were sampled in six permanently open estuaries along the south coast of New South Wales, Australia. These included three heavily modified estuaries, Port Jackson (33°44.258′S, 151°16.542′E), Botany Bay (33°59.352′S, 151°11.433′E), and Port Kembla (34°28.121′S, 150°54.410′E), and three relatively unmodified estuaries, Port Hacking (34°04.680′S, 151°09.311′E), Jervis Bay (35°04.762′S, 150°44.858′E), and the Clyde River (35°44.233′S, 150°14.272′E) (Figure 1). The three heavily modified estuaries are all anthropogenically disturbed environments close to large urban and industrial areas and are subject to intense commercial and recreational boating traffic, historic and ongoing contamination, concentrated recreational fishing activity, frequent dredging for navigation and construction, and substantial urbanization of their shoreline and catchment. In comparison, the relatively unmodified estuaries have less concentrated fishing activity, less boating traffic (almost none of which is commercial), less urbanization of the coastline and catchment, and virtually no heavy industry [22], [23]. While these estuaries do have some degree of agricultural land use in their catchment, the majority of the catchment in all of the relatively unmodified estuaries is within conservation areas, forestry zones, or areas where anthropogenic utilization is negligible [24]. Both the Clyde River (within Bateman's Bay Marine Park) and Jervis Bay (Jervis Bay Marine Park) are within marine parks. Port Hacking is located between the suburbs of southern Sydney and the forested slopes of Royal National Park, which lines the southern border of the estuary. While not strictly within a marine park, Port Hacking's catchment is largely intact due to its proximity to the Royal National Park and there is no major industrial activity within the estuary, though navigation channels in the outer zone are periodically dredged [23]. Previous monitoring indicates that the heavily modified estuaries are also nutrient enriched, whilst nutrient levels in the relatively unmodified estuaries are less elevated [22].

Each estuary was divided into an inner and outer zone (see site coding in Figure 1) based on qualitative observations of physical characteristics. The inner zone is further away from the ocean and represents the lower reaches of the estuarine tributary. In this zone turbidity and temperatures are generally higher than in the outer zone [13]. The outer zone sites are near the marine entrance to the estuaries where salinity, coastal flushing, wave exposure, and oceanic current systems have greater influence. In this zone sediment grain sizes are also larger and there is greater tidal influence [13]. While all of the estuaries examined in this study are permanently open tidal systems, estuary size varies. As such, estuary size is included in our analysis as a measure of structural variability.

It should also be noted that Jervis Bay and the Clyde River are within a different bioregion than the other four estuaries, according to the Interim Biogeographic Regionalization of Australia (IBRA) system, though the maximum distance between estuaries is only 275 km (Batemans Bay to Port Jackson) [25]. While this indicates that some differences exist in the biological and environmental conditions between these areas, most of the fish species examined in this study are known to occur in all the estuaries examined in this study. Notably, the six estuaries examined in this study are at least several hundred kilometers within the known range of the major species which drive the trends in this analysis [26]. In addition, differences in physico-chemical variables between zones were greater than between estuaries and the habitat sampled was judged to be reasonably similar in all estuaries (beaches). For these reasons, we believe that comparisons between these estuaries are valid for this analysis, despite different bioregional classification. Other studies have utilized similar comparisons between these estuaries [4], [9].

### Fish Sampling Methods

Within each estuary six beach sites consisting of bare sediment were selected on an *ad hoc* basis, with three sites in each zone. In order to capture temporal variability in these physico-chemical conditions, we conducted four sampling rounds over two years. Thus, there were a total of four rounds of sampling; November–December 2009 (Early Summer), February–March 2010 (Summer), November–December 2010 (Early Summer) and February–March 2011 (Summer). On the south-east coast of Australia, the “Summer” season coincides with the warmest ocean water temperatures. Fish were sampled using a beach seine net with a 20 m headline, a 2 m drop, a 1.5 m cod end, constructed from a 12 mm mesh. Before deployment a 10 m segment was measured out on each beach and the net was pulled out straight for 10 m prior to encircling the beach. In this way an area of approximately 100 m^2^ was sampled at each time of sampling. Only sites where the researchers could pull the beach seine out 10 m from shore while keeping their head above water were sampled. As such, all sites had a shallow slope (slope ≤−0.2) and the sampling depth was 1–1.8 m (at its deepest point, 10 m from shore). All fish were euthanized using a 100 mg L^−1^ benzocaine solution and frozen for transportation back to the laboratory. Fish were sorted to species and standard length and wet weight measurements were taken. Due to identification difficulties, *Sillago* sp. (whiting) <10 cm in length were classified to genus only. Individuals larger than this were identified as either *Sillago maculata* or *Sillago ciliata*.

### Physico-chemical Sampling Methods

At each sampling time and location physico-chemical data (temperature, salinity, pH, turbidity) were collected using a YSI-Sonde 6820-V2 (Yellow Springs, USA) (calibrated weekly). At each site benthic sediments were collected once at 5 m depth between Feb–Mar 2010 using a sediment grab. Sediments used for metal analyses were oven dried at 50°C before being homogenized to a fine powder in a ball mill. A 0.5 g sub-sample from each site was digested according to Method 3051A [27]. Specifically, the sediments were digested in 9 mL HNO_3_ and 3 mL HCl for 20 min at 200°C in a 1000 W microwave. Following digestion, samples were diluted up to 30 mL with Milli-Q water and analyzed for metal content (As, Co, Cr, Cu, Fe, Mn, Ni, Pb, Zn) using inductively coupled plasma-optical emission spectroscopy (ICP-OES). The instrument was calibrated with matrix-matched standards and had limits of detection (LOD) of 3 mg kg^−1^ for Cd, Co, Mn and Zn, and 5–25 mg kg^−1^ for As, Cu, Ni and Pb. Analysis of certified reference materials (sediment CRM – LGC6137 and oyster CRM – 1566b, Graham B. Jackson Pty Ltd, Australia) indicated adequate recoveries (within ±15%) for most metals. Where recoveries were outside this range, the data were omitted from analysis. Outer zone sites were primarily sandy and so only inner zone sites of each estuary were analyzed for organic contaminants (all sites were analyzed for metals). Samples were analyzed for 16 priority PAHs: naphthalene, acenaphthylene, acenaphthene, fluorene, phenanthrene, anthracene, fluoranthene, pyrene, benz(a)anthracene, chrysene, benzo(a)pyrene, benzo(b)fluoranthene, benzo(k)fluoranthene, indeno(1,2,3-cd)pyrene, dibenz(a,h)anthracene and benzo(g,h,i)perylene by the National Measurement Institute (Sydney, Australia). Values were then normalized to 1% total organic carbon for comparison with sediment quality guidelines.

Sediments were selected to measure contaminants in these systems (rather than a water column measure of contamination) for several reasons. First, it is well known that fish accumulate contaminants through their food to a much greater degree than through their gills or through interaction with contaminated water [28], [29]. The majority of species in this study are benthic or benthopelagic foragers and so most would interact with sediments regularly during feeding [17]. Second, contaminants accumulate in estuarine sediments over the long-term, as such sediment metals values are less temporally variable and represent a contemporary threat from historical pollution sources [30].

For univariate analysis and graphical presentation of total metals and PAH contamination a combined sediment metals and PAH quotient were calculated following Long et al. (2006) [31]. Each individual metal contaminant load was divided by the low and high trigger values from the ANZECC sediment quality guidelines [32]. The high and low quotients for each contaminant were then summed for each sample and divided by two to give a mean sediment quality guideline quotient (mSQGQ) for each sample. The ANZECC trigger values are threshold values which are meant to provide a baseline for assessing the impacts of marine and freshwater contaminants. Where concentrations of contaminants exceed the trigger values, it is believed that there is a risk of adverse environmental effects [32].

### Ecological Classification

In order to evaluate the role of ecological characteristics in determining the relative sensitivity of different functional groups, species were classified both according to their trophic level and their usage of estuarine environments during their life cycle (Appendix S1). The numeric trophic level of each species was determined using the fish database website *Fishbase* [33] and this data was used to calculate a Marine Trophic Index for each sample [34]. In addition, each species was classified into discrete guilds based on their usage of estuaries during their life cycle following Elliot *et al.* (2007). Three broad categories were used in this study:

- Estuarine Species: Species which spawn within the estuary and which normally complete their entire life cycle within the estuarine environment.
- Estuarine Opportunists: Species which primarily spawn in marine coastal waters but enter the estuary either in their larvae or juvenile stages. Many of these species require sheltered estuarine habitats during their larval and juvenile stages and are hence dependent on the estuarine environments for reproduction. Most species spend part of their adult stage outside of estuaries.
- Marine Stragglers: Species which spawn at sea and normally enter estuaries only in low numbers, occurring most frequently in the lower reaches of the estuary. Many are stenohaline and are primarily associated with coastal marine waters.

These classifications were made through a review of existing literature for these species [10], [35], [36] and through consultation with regional fish experts (A.G. Miskiewicz, personal communication, 2011).

### Statistical Analysis

All multivariate and univariate datasets were analyzed as mixed-model PERMANOVA in PRIMER v.6.4 [37]. Prior to analysis, abundance and biomass data were log(*x*+1) transformed. Bray-Curtis similarity matrices were calculated for multivariate data while Euclidean similarity matrices were used for univariate measures. A dummy variable of 1 was added when calculating the similarity matrices in order to compensate for zero values. The PERMANOVA design employed in the course of this analysis consisted of the following factors:

Dis - Disturbance category – Heavily Modified or Relatively Unmodified (2 levels, Fixed)

Zo - Zone – Inner or Outer (2 levels, Fixed)

Ti – Time of Year – Early Summer or Summer (2 levels, Random)

Ye – Year – 2009–2010 or 2010–2011 (2 levels, Random)

Es – Estuary (Disturbance Category) – (6 estuaries, Random)

Si – Site (Estuary(Disturbance Category)×Zone) – (36 sites, Random)

Reduced versions of this model were used to analyze the sediment metals and PAH data. In these reduced models the Time and Year factors were removed as these covariates were not replicated. Monte-Carlo p-values were used in some places where the number of unique permutations was less than 20 (these values are marked in tables) [37]. Analysis of covariation of physico-chemical, metal, and PAH covariates was conducted using the distance-based linear model (DistLM) in PERMANOVA. This program calculates a distance-based multivariate multiple regression (e.g. dbRDA) for any linear model on the basis of any distance measure, using permutation procedures [38]. The ‘Best’ selection procedure was employed using BIC selection criteria. Covariate factors were then analyzed graphically using Principal Coordinated Ordination (PCO). PCO is a PERMANOVA function that performs a principal coordinate analysis of any symmetric distance matrix. This analysis is also called metric multi-dimensional scaling [39]. All covariate factors were plotted in the PCO charts, however, turbidity did not correlate strongly enough to show a discernable vector line. The highest correlating species (those with a multiple correlation factor >0.2) were also included in the PCO charts.

## Results

### Fish Assemblage Characteristics

In total more than 10,350 fish representing 51 species were collected and identified during the study. Thirty of these species were relatively rare, represented by only 1–10 individuals over the course of the study. By abundance the 10 most common species accounted for ∼92.5% of the fish assemblage. In order of abundance these were *Ambassis jacksoniensis* (39.8%), *Myxus elongatus* (18.7%), *Sillago* sp. (9.3%), *Leptatherina presbyteroides* (7.8%), *Hyperlophus vittatus* (3.7%), *Gerres subfasciatus* (3.2%), *Atherinomorus vaigiensis* (3.1%), *Favonigobius lentiginosus* (2.8%), *Sillago maculata* (2.3%), and *Torquigener pleurogramma* (1.9%). The summarized biological dataset can be found in Appendix S1.

### Physico-chemical variables and Sediment Contamination

**Figure 2:**
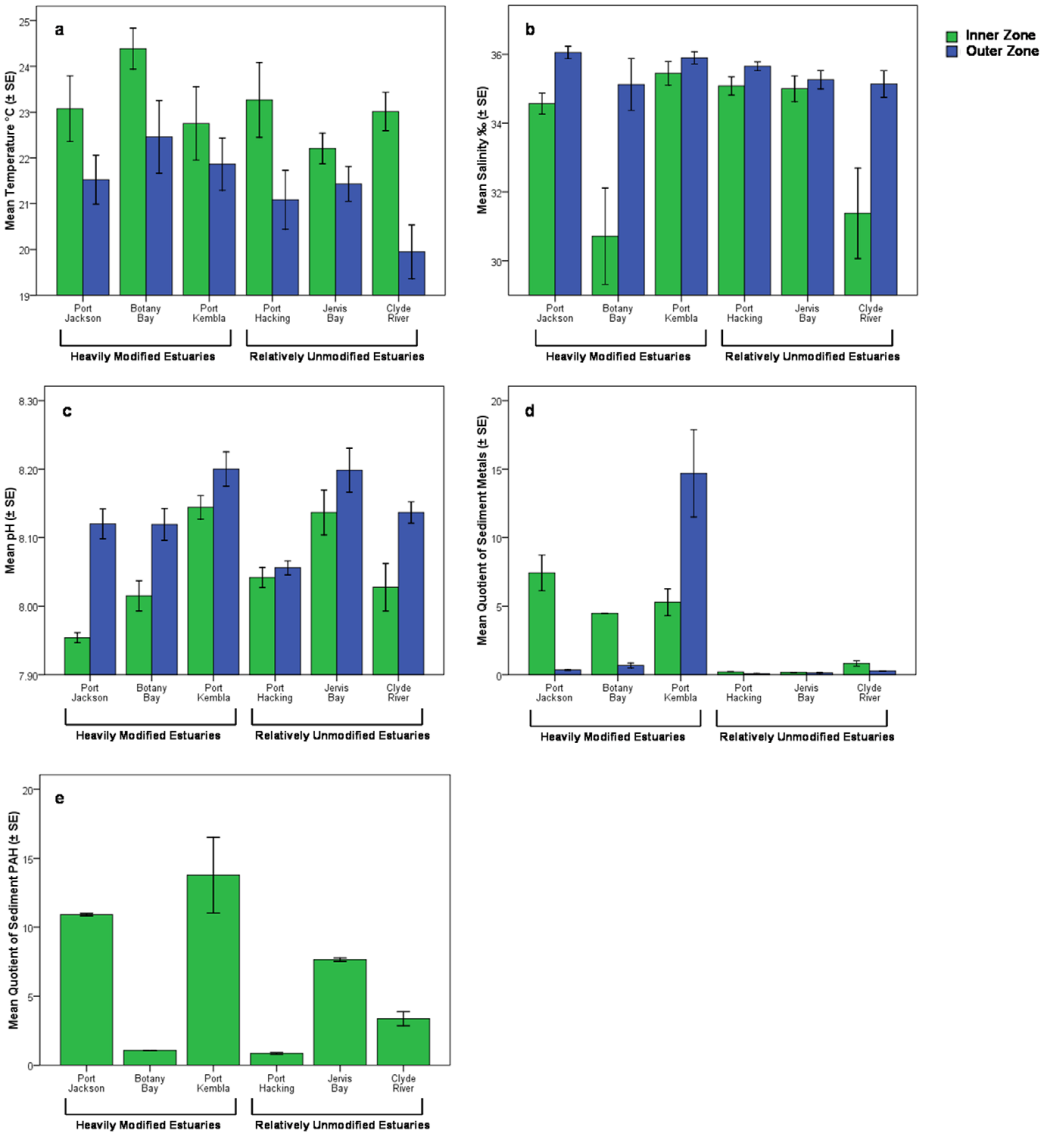
Mean (±SE) physico-chemical and sediment contaminant variables by zone and estuary. Including a) Temperature, b) Salinity, c) pH, d) Mean Quotient of Sediment Metals, and e) Mean Quotient of Sediment PAH values.

In most estuaries, temperature (Figure 2a) was higher in the inner zone sites while salinity (Figure 2b) and pH (Figure 2c) were lower in the inner zone (Table 1 a–c). Physico-chemical variables also showed significant temporal variation (Table 1, Ye×Ti). While this variation was significant, the trends in physico-chemical variables between zones remained consistent. Turbidity did not show a significant trend by zone (Table 1d). There appeared to be higher concentrations of sediment metals in the inner zones of the heavily modified estuaries as well as the outer zone of the heavily modified Port Kembla (Figure 2d). This resulted in a significant interaction between zone and estuary nested within disturbance category (Table 2, 3a). The outer zones of all other estuaries (heavily modified or relatively unmodified) and the inner zone of the relatively unmodified estuaries displayed much lower levels of sediment metal contamination (Figure 2d). PAH contamination was only measured in the inner zones and did not show a clear trend by disturbance category, but did differ by estuary (Table 3b). The heavily modified estuaries Port Jackson and Port Kembla displayed relatively high PAH values (Table 1c, Figure 2e). Sediment metals values at many of the inner zones within the heavily modified estuaries and in the outer zone of Port Kembla were above levels predicted to have biological effects on infauna according to water quality guidelines [32].

**Table 1:** Univariate analysis of physico-chemical variables and estuary size covariates.

		a) Temperature			b) Salinity			c) pH			d) Turbidity		
Source	dF	MS	F	p-value	MS	F	p-value	MS	F	p-value	MS	F	p-value
Dis	1	4.84	1.71	0.250	0.01	0.28	0.961	0.17	0.61	0.738	0.00	0.45	0.944
Zo	1	20.05	6.71	**0.004**	16.34	3.79	**0.038**	23.25	10.63	**0.004**	0.74	0.90	0.557
Ye	1	7.83	0.34	0.835	0.01	0.14	0.974	8.70	2.44	0.194	0.02	0.38	0.890
Ti	1	26.87	1.00	0.517	5.93	0.36	0.844	1.68	0.83	0.557	2.25	1.29	0.376
Es(Dis)	4	1.20	0.73	0.797	5.86	3.62	**0.001**	9.39	3.11	**0.001**	3.81	1.45	0.089
DisxZo	1	0.51	0.64	0.713	0.42	0.18	0.988	1.77	0.67	0.703	6.46	2.40	0.061
DisxYe	1	0.11	0.86	0.530	0.16	3.31	0.133	8.06	0.80	0.594	1.67	1.36	0.358
DisxTi	1	2.79	1.11	0.468	0.21	1.72	0.306	3.19	0.41	0.803	0.51	1.34	0.368
ZoxYe	1	0.57	2.43	0.217	0.37	0.32	0.876	0.61	1.65	0.316	2.02	1.14	0.434
ZoxTi	1	0.80	1.48	0.362	0.81	0.37	0.831	0.08	1.82	0.275	0.12	0.44	0.821
YexTi	1	25.35	25.88	0.007	24.98	7.10	0.048	2.45	1.58	0.285	1.74	1.26	0.350
ZoxEs(Dis)	4	0.90	1.67	0.078	3.26	4.46	0.001	1.42	1.93	0.042	1.49	0.85	0.738
Es(Dis)xYe	4	0.41	0.38	0.815	0.52	0.18	0.938	1.75	1.12	0.378	1.91	1.16	0.382
Es(Dis)xTi	4	2.56	2.36	0.091	1.58	0.47	0.747	1.43	0.98	0.443	1.07	0.84	0.563
DisxZoxYe	1	0.15	9.03	0.035	0.30	6.98	0.030	1.77	3.34	0.146	1.69	0.81	0.620
DisxZoxTi	1	0.06	1.47	0.367	1.05	4.56	0.057	0.31	1.84	0.296	0.47	0.52	0.788
DisxYexTi	1	0.85	0.87	0.402	0.59	0.17	0.708	10.25	6.61	0.066	0.34	0.25	0.740
ZoxYexTi	1	0.49	0.54	0.501	6.22	3.74	0.127	0.03	0.21	0.694	0.55	1.17	0.349
Si(Es(Dis)xZo)	24	0.46	1.46	0.107	0.42	2.93	0.006	0.25	1.71	0.034	0.97	1.11	0.248
ZoxEs(Dis)xYe	4	0.12	0.15	1.000	0.14	0.15	1.000	0.42	1.97	0.104	1.63	1.76	0.065
ZoxEs(Dis)xTi	4	0.66	0.69	0.793	0.46	0.33	0.986	0.09	1.22	0.341	0.78	1.28	0.264
Es(Dis)xYexTi	4	0.98	18.34	0.001	3.52	29.69	0.001	1.55	9.47	0.001	1.38	2.18	0.032
DisxZoxYexTi	1	0.00	0.00	0.952	0.14	0.08	0.774	0.15	1.12	0.355	1.03	2.19	0.193
YexSi(Es(Dis)xZo)	24	0.23	4.27	0.001	0.08	0.70	0.796	0.16	1.01	0.517	0.82	1.29	0.149
TixSi(Es(Dis)xZo)	24	0.12	2.33	0.026	0.10	0.86	0.657	0.08	0.48	0.957	0.63	1.00	0.495
ZoxEs(Dis)xYexTi	4	0.91	17.07	0.001	1.66	14.05	0.001	0.13	0.81	0.539	0.47	0.74	0.665
Res	24	0.05			0.12			0.16			0.63		

a) Temperature, b) Salinity, c) pH, and d) Turbidity. Factors: Dis = Disturbance Category (Heavily Modified vs. Relatively Unmodified), Zo = Zone (Inner vs. Outer), Ti = Time of Sampling, Ye = Year, Es = Estuary, Si = Site. Bold values correspond to significant values for higher-level factors or interactions between non-random factors.

**Table 2:** Univariate analysis of sediment metals quotient in the full model.

Metals Quotient - Full Model				
Source	dF	MS	F	p-value
Dis	1	32.05	5.18	*0.084
Zo	1	0.15	0.02	0.926
Es(Dis)	4	6.18	2.65	**0.032**
DisxZo	1	0.02	0.00	0.987
Es(Dis)xZo	4	7.52	3.23	**0.014**
Si(Es(Dis)xZo)	24	2.33	Den = 0	
Res	108	0.00		

Factors: Dis = Disturbance Category (Heavily Modified vs. Relatively Unmodified), Zo = Zone (Inner vs. Outer), Es = Estuary, Si = Site. Bold values correspond to significant values for higher-level factors or interactions between non-random factors.

*Indicates Monte Carlo p value.

**Table 3:** Univariate analysis of a) sediment metals quotient and b) sediment PAH quotient under a reduced model (inner zone only).

		a) Metal Quotient - Inner Only			b) PAH Quotient - Inner Only		
Source	dF	MS	F	p-value	MS	F	p-value
Dis	1	39.13	34.95	* **0.006**	9.89	1.14	*0.349
Es(Dis)	4	1.12	0.49	0.795	8.65	3.91	**0.029**
Si(Es(Dis))	12	2.28	Den = 0		2.21	Den = 0	
Res	54	0.00			0.00		

Factors: Dis = Disturbance Category (Heavily Modified vs. Relatively Unmodified), Es = Estuary, Si = Site. Bold values correspond to significant values for higher-level factors or interactions between non-random factors.

*Indicates Monte Carlo p value.

In some analyses several of the random interaction terms were also significantly different (e.g. Ti, Ye, Si(Es(Dis)xZo) and Es(Dis)). Here and elsewhere, the test of the main effects are still considered, as higher level fixed-factor effects remain relevant despite an interaction with a random factor [40].

### Fish Assemblages – Within Estuary Variation

**Figure 3:**
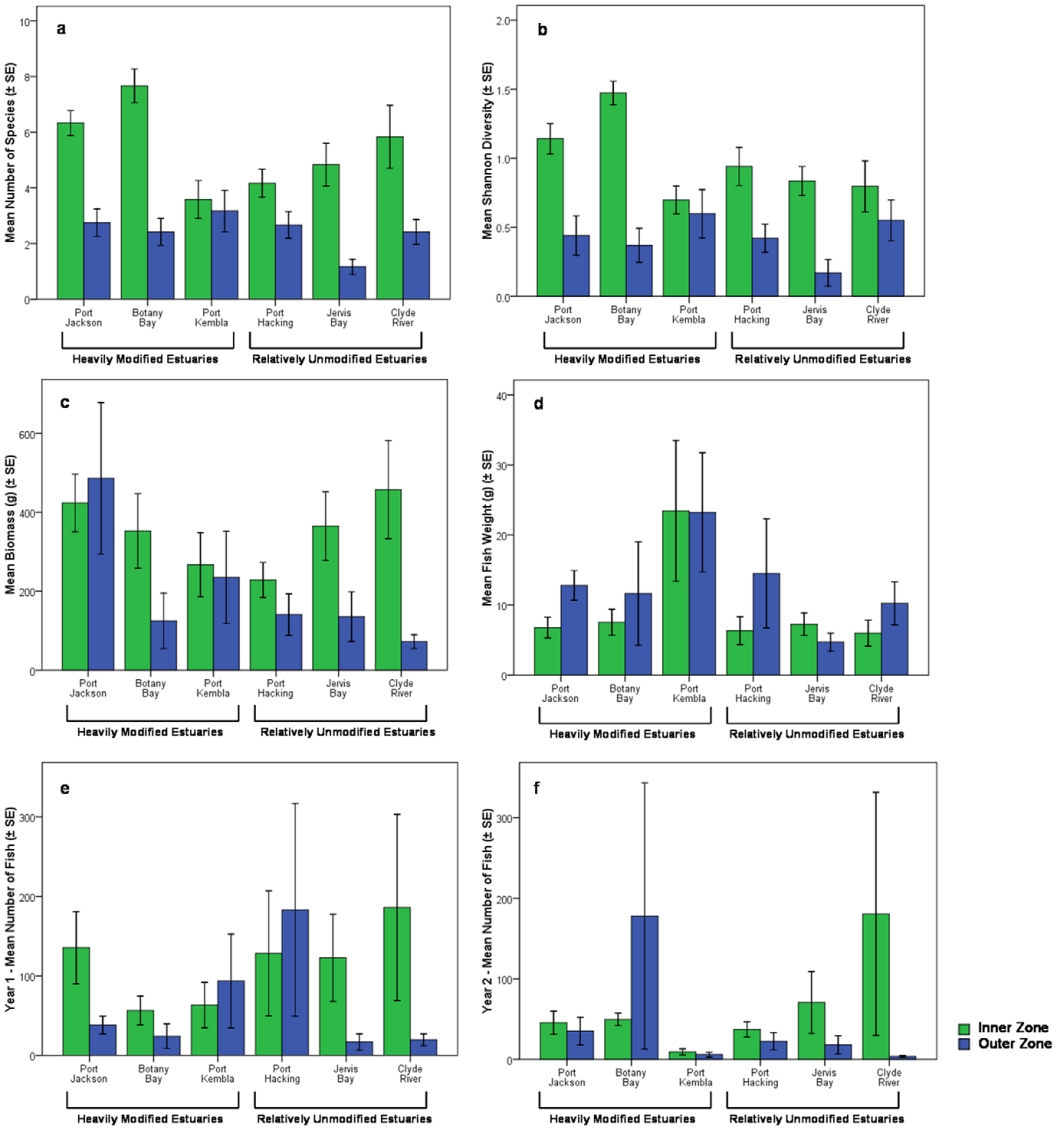
Mean (±SE) community level indicators by zone/estuary. Including a) Species Richness, b) Shannon Diversity, c) Biomass, d) Average Fish Weight, e) Year 1 Abundance, f) Year 2 Abundance.

Species richness, Shannon diversity, and fish biomass were all significantly greater in the inner zones compared to the outer zones (Table 4 Figure 3a,b,c), while no main effects or interaction terms were detected for any of these measures for disturbance category (Table 4). Species richness and Shannon diversity also showed significant variation by site (Table 4a,b). Average fish weight was greater in the outer zones of all estuaries except Port Kembla and Jervis Bay, where the average fish weight was approximately equal between zones (Table 4, Figure 3d). Port Jackson displayed a trend towards having higher fish biomass than other estuaries across both zones (Figure 3c) while Port Kembla displayed a trend towards higher average fish weight than other estuaries (Figure 3d). The pattern of increased biomass, species richness and Shannon diversity in the inner estuary was weakest for Port Kembla, where we also observed the smallest difference between zones for physico-chemical variables. Interestingly, the outer harbor of Port Kembla was the only outer zone to contain substantial sediment contamination but this did not relate to reduced fish biomass, species richness, or Shannon diversity relative to other outer zones.

**Table 4:** Univariate analysis of a) Species Richness, b) Shannon Diversity, c) Biomass, d) Average Fish Weight, and e) Abundance.

		a) Species Richness			b) Shannon Diversity			c) Biomass			d) Fish Size			e) Abundance		
Source	dF	MS	F	p-value	MS	F	p-value	MS	F	p-value	MS	F	p-value	MS	F	p-value
Dis	1	23.36	3.08	0.079	1.01	2.12	0.170	6.17	2.85	0.080	497.02	1.10	0.463	0.03	2.25	0.136
Zo	1	318.03	10.47	**0.005**	11.12	10.75	**0.003**	66.32	4.31	**0.032**	660.14	5.75	**0.011**	53.08	7.51	**0.006**
Ye	1	17.36	1.16	0.432	0.08	0.24	0.894	4.65	1.14	0.467	738.15	1.09	0.469	21.44	10.82	**0.015**
Ti	1	0.69	0.18	0.943	0.10	0.28	0.878	0.77	0.63	0.693	39.37	0.56	0.506	0.81	3.23	0.139
Es(Dis)	4	12.66	0.85	0.674	0.35	0.64	0.883	5.22	0.99	0.508	526.49	0.79	0.714	5.08	1.61	0.108
DisxZo	1	0.44	0.41	0.884	0.22	0.54	0.777	4.70	0.68	0.679	0.81	0.40	0.883	2.91	1.20	0.411
DisxYe	1	0.11	0.10	0.982	0.15	0.78	0.587	0.20	0.14	0.959	528.89	0.82	0.562	0.21	0.18	0.944
DisxTi	1	1.00	0.17	0.945	0.15	0.94	0.494	0.18	0.16	0.950	270.78	1.36	0.317	0.03	0.18	0.946
ZoxYe	1	5.44	1.81	0.306	0.00	0.36	0.811	0.00	3.36	0.138	47.80	0.26	0.885	0.00	0.17	0.938
ZoxTi	1	1.00	0.52	0.726	0.00	0.35	0.831	5.47	3.14	0.157	10.37	0.15	0.976	4.35	0.79	0.561
YexTi	1	9.00	4.97	0.098	0.54	4.07	0.135	2.33	1.49	0.286	5.57	0.05	0.812	0.01	0.00	0.962
ZoxEs(Dis)	4	22.30	1.34	0.219	0.90	1.68	0.069	5.72	1.56	0.112	114.94	0.80	0.705	2.90	1.08	0.435
Es(Dis)xYe	4	7.59	2.52	0.075	0.33	2.27	0.093	3.12	1.00	0.417	810.73	2.51	0.064	2.23	0.65	0.621
Es(Dis)xTi	4	4.74	1.37	0.281	0.28	1.54	0.225	1.39	1.03	0.407	287.14	1.28	0.324	1.12	0.53	0.736
DisxZoxYe	1	0.69	0.80	0.568	0.15	1.03	0.483	0.17	7.59	0.040	26.21	0.41	0.809	0.90	2.17	0.248
DisxZoxTi	1	2.25	0.73	0.635	0.05	0.69	0.604	0.01	1.99	0.255	926.89	3.74	0.108	0.61	0.53	0.720
DisxYexTi	1	12.25	6.76	0.060	0.02	0.16	0.701	9.78	6.24	0.092	8.48	0.07	0.777	14.91	5.29	0.098
ZoxYexTi	1	0.25	0.09	0.772	0.42	2.16	0.219	1.28	0.26	0.635	198.57	3.16	0.131	3.98	5.08	0.086
Si(Es(Dis)xZo)	24	9.20	1.90	0.016	0.36	2.02	0.006	6.11	1.39	0.122	286.73	0.81	0.720	2.96	0.97	0.552
ZoxEs(Dis)xYe	4	4.26	1.44	0.219	0.12	1.05	0.469	0.21	0.29	0.987	197.69	1.26	0.336	0.69	0.59	0.855
ZoxEs(Dis)xTi	4	6.85	1.49	0.218	0.14	0.91	0.594	2.06	0.62	0.830	259.05	1.43	0.279	2.54	1.50	0.221
Es(Dis)xYexTi	4	1.81	0.54	0.703	0.13	0.69	0.590	1.57	0.65	0.631	110.07	0.41	0.803	2.82	2.16	0.097
DisxZoxYexTi	1	0.00	0.00	1.000	0.21	1.10	0.342	0.47	0.09	0.782	11.67	0.19	0.703	0.09	0.11	0.740
YexSi(Es(Dis)xZo)	24	2.52	0.76	0.766	0.10	0.52	0.937	3.97	1.64	0.109	344.63	1.27	0.328	2.61	2.00	0.046
TixSi(Es(Dis)xZo)	24	4.09	1.23	0.331	0.17	0.91	0.579	2.15	0.89	0.627	342.85	1.26	0.339	1.78	1.36	0.212
ZoxEs(Dis)xYexTi	4	2.73	0.82	0.533	0.19	1.01	0.431	5.03	2.08	0.125	49.70	0.18	0.944	0.78	0.60	0.650
Res	24	3.33			0.19			2.42			271.34			1.30		

Factors: Dis = Disturbance Category (Heavily Modified vs. Relatively Unmodified), Zo = Zone (Inner vs. Outer), Ti = Time of Sampling, Ye = Year, Es = Estuary, Si = Site. Bold values correspond to significant values for higher-level factors or interactions between non-random factors.

**Figure 4:**
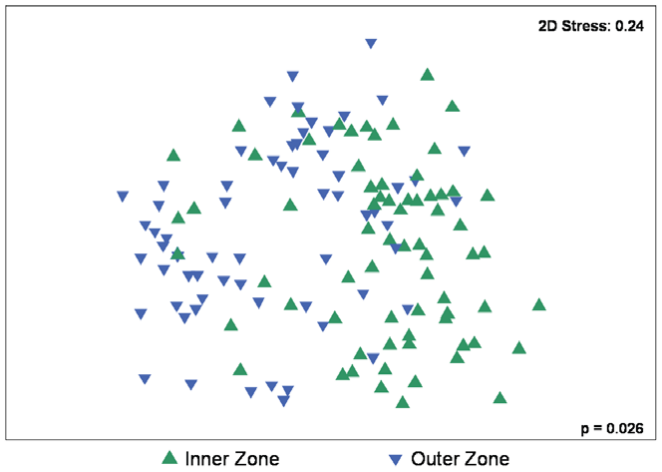
Two dimensional MDS plot of multivariate assemblage composition by zone. Symbols represent centroids of the assemblage composition. Stress value of 0.24 represents a relatively weak ordination of the multivariate data, which is not the result of dispersion (p = 0.625).

**Figure 5:**
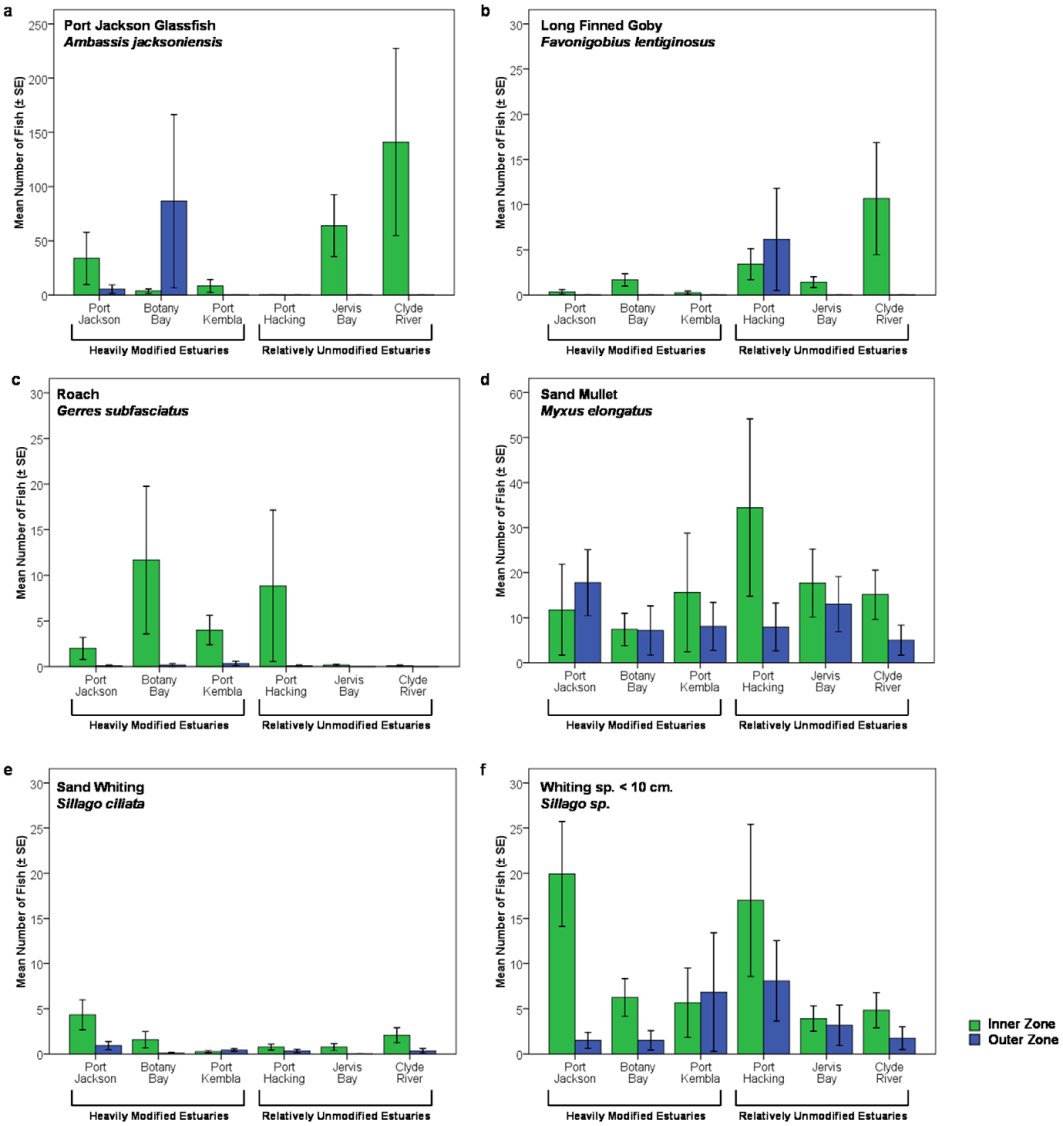
Mean (±SE) abundance by zone/estuary for 100 m^2^ beach seine samples. Plots of top six species contributing to differences between inner and outer zones.

Fish abundance was significantly higher in the inner zones and in the first year of sampling (Table 4, Figure 3 e,f). While fish abundance was significantly greater in the inner zone overall, occasionally the difference between zones was small, and Botany Bay appeared to have a greater abundance in the outer zone during year 2 (Figure 3 e,f). Multivariate analysis of the community composition found that inner and outer zone fish communities differed significantly (Table 5, Figure 4). There was also significant variation in community composition between sites (Table 5). Simper analysis revealed that the top six species contributing to differences between zones were *M. elongatus*, *Sillago* sp. (<10 cm), *A. jacksoniensis*, *S. ciliata*, *G. subfasciatus*, and *F. lentiginosus*. All of these species were more abundant in the inner zone and collectively they contributed to approximately 59% of the difference between zones. However, several of these species also varied significantly by other factors. Differences in abundance between zones were significant for the *Sillago* sp., *S. ciliata*, *G. subfasciatus*, *F. lentiginosus*, and nearly significant for *A. jacksoniensis* (Table 6 a–f, Figure 5). *F. lentiginosus*, *S. ciliata*, and *Sillago sp*. also varied significantly by site. *A. jacksoniensis* showed a significant zone×estuary interaction, which was driven by higher abundances in the inner zone for all estuaries except Botany Bay, where it was more abundant in the outer zone. Of these species, only *G. subfasciatus* differed by disturbance category with significantly more individuals in the inner zone of the heavily modified estuaries (Table 6c). This resulted in a near-significant zone×disturbance category interaction.

**Table 5:** Multivariate analysis of community composition in the full model.

Community Composition - Full Model				
Source	dF	MS	F	p-value
Dis	1	6513.20	1.28	0.269
Zo	1	32951.00	3.85	**0.026**
Ye	1	5943.40	1.49	0.238
Ti	1	1815.80	0.75	0.666
Es(Dis)	4	4793.30	1.20	0.088
DisxZo	1	5344.70	0.87	0.627
DisxYe	1	1468.40	0.66	0.768
DisxTi	1	2400.90	0.83	0.614
ZoxYe	1	2451.00	1.29	0.321
ZoxTi	1	1716.60	0.90	0.576
YexTi	1	3692.20	1.61	0.229
ZoxEs(Dis)	4	3732.20	1.14	0.182
Es(Dis)xYe	4	1824.20	0.84	0.700
Es(Dis)xTi	4	1790.20	0.82	0.685
DisxZoxYe	1	2140.60	2.82	0.033
DisxZoxTi	1	2036.10	1.89	0.144
DisxYexTi	1	3891.70	1.69	0.196
ZoxYexTi	1	2337.40	1.16	0.333
Si(Es(Dis)xZo)	24	3625.40	1.48	0.001
ZoxEs(Dis)xYe	4	1127.00	0.71	0.955
ZoxEs(Dis)xTi	4	1798.50	0.88	0.728
Es(Dis)xYexTi	4	2300.40	1.49	0.056
DisxZoxYexTi	1	347.56	0.17	0.938
YexSi(Es(Dis)xZo)	24	1720.80	1.12	0.243
TixSi(Es(Dis)xZo)	24	1763.80	1.15	0.218
ZoxEs(Dis)xYexTi	4	2021.40	1.31	0.139
Res	24	1540.20		

Factors: Dis = Disturbance Category (Heavily Modified vs. Relatively Unmodified), Zo = Zone (Inner vs. Outer), Ti = Time of Sampling, Ye = Year, Es = Estuary, Si = Site. Bold values correspond to significant values for higher-level factors or interactions between non-random factors.

**Table 6:** Univariate analysis of the abundance of the top six species contributing to differences between zones a) Ambassis jacksoniensis, b) Favonigobius lentiginosus, c) Gerres subfasciatus, d) Myxus elongatus, e) Sillago ciliata, and f) Sillago sp.

		a) A. jacksoniensis			b) F. lentiginosus			c) G. subfasciatus			d) M. elongatus			e) S. ciliata			f) Sillago sp.		
Source	dF	MS	F	p-value	MS	F	p-value	MS	F	p-value	MS	F	p-value	MS	F	p-value	MS	F	p-value
Dis	1	0.39	0.70	0.687	3.82	1.75	0.216	6.51	4.75	**0.028**	6.41	2.09	0.151	0.70	0.64	0.728	0.06	0.34	0.926
Zo	1	33.86	3.16	**0.058**	8.91	9.56	**0.002**	12.07	4.59	**0.028**	4.39	1.28	0.387	5.81	3.56	**0.049**	30.26	5.61	**0.015**
Ye	1	1.16	0.36	0.833	0.62	1.29	0.419	0.00	0.31	0.871	6.85	3.98	0.104	0.17	0.48	0.753	3.99	3.04	0.159
Ti	1	2.47	0.44	0.798	0.07	1.67	0.328	3.68	3.87	0.115	2.34	1.78	0.322	0.55	0.96	0.503	0.01	1.12	0.459
Es(Dis)	4	6.13	1.83	0.059	0.66	0.84	0.661	1.01	1.01	0.493	0.65	0.93	0.590	1.48	1.50	0.137	3.32	1.09	0.424
DisxZo	1	10.78	1.63	0.265	0.86	1.96	0.170	4.09	3.69	0.051	10.92	1.15	0.435	0.00	0.25	0.964	2.29	0.82	0.562
DisxYe	1	0.11	0.45	0.764	0.56	1.49	0.378	0.19	2.56	0.191	0.01	0.25	0.893	0.23	1.52	0.378	0.96	0.74	0.591
DisxTi	1	1.32	0.54	0.716	0.80	5.08	0.071	0.22	0.47	0.770	7.75	0.64	0.660	0.00	1.28	0.439	2.10	3.00	0.160
ZoxYe	1	3.96	0.74	0.601	0.16	0.29	0.894	0.03	0.21	0.923	1.23	1.75	0.307	0.17	0.50	0.740	2.95	7.33	0.039
ZoxTi	1	2.10	0.45	0.765	0.01	0.63	0.677	1.93	1.74	0.291	0.45	0.77	0.623	0.55	0.98	0.517	0.03	0.49	0.770
YexTi	1	5.85	4.54	0.093	0.34	0.45	0.544	0.29	2.39	0.197	1.34	0.29	0.603	0.66	2.07	0.228	0.07	0.16	0.715
ZoxEs(Dis)	4	6.05	2.04	0.036	0.69	0.56	0.959	0.80	0.96	0.528	2.55	1.08	0.406	0.83	1.36	0.195	2.02	0.88	0.631
Es(Dis)xYe	4	0.92	0.87	0.514	0.73	1.10	0.379	0.10	0.78	0.584	1.56	0.44	0.778	0.36	1.19	0.337	1.39	1.20	0.339
Es(Dis)xTi	4	2.71	0.76	0.562	0.16	0.90	0.496	0.69	1.70	0.172	2.61	0.58	0.685	0.25	0.88	0.522	0.34	1.49	0.250
DisxZoxYe	1	0.33	0.64	0.687	0.14	0.23	0.910	0.26	1.20	0.403	0.32	4.88	0.076	0.01	1.41	0.351	1.40	3.25	0.132
DisxZoxTi	1	1.62	0.85	0.560	0.27	0.84	0.541	0.03	0.71	0.628	6.56	4.74	0.079	0.38	7.43	0.033	1.25	1.14	0.454
DisxYexTi	1	2.17	1.68	0.259	0.15	0.20	0.667	0.02	0.18	0.682	16.83	3.60	0.132	0.00	0.00	0.951	0.51	1.14	0.340
ZoxYexTi	1	6.34	4.06	0.124	0.20	0.92	0.418	0.87	3.69	0.121	1.44	0.72	0.433	0.96	2.03	0.202	0.10	0.13	0.742
Si(Es(Dis)xZo)	24	2.12	0.62	0.964	1.00	2.22	0.012	0.92	1.34	0.142	4.39	1.47	0.120	0.64	1.96	0.017	2.65	1.66	0.047
ZoxEs(Dis)xYe	4	1.08	0.84	0.637	1.13	2.39	0.045	0.41	1.10	0.393	0.40	0.44	0.943	0.31	0.84	0.654	0.41	0.65	0.778
ZoxEs(Dis)xTi	4	1.87	0.59	0.852	0.17	2.05	0.076	0.37	0.99	0.481	1.73	0.75	0.716	0.08	0.46	0.926	1.50	2.02	0.100
Es(Dis)xYexTi	4	1.29	0.79	0.562	0.76	1.06	0.390	0.12	0.34	0.855	4.68	3.66	0.019	0.32	1.22	0.327	0.45	0.37	0.811
DisxZoxYexTi	1	1.89	1.21	0.340	0.41	1.89	0.243	0.00	0.00	0.975	0.07	0.04	0.854	0.04	0.08	0.784	0.25	0.34	0.609
YexSi(Es(Dis)xZo)	24	1.65	1.02	0.502	0.56	0.78	0.763	0.46	1.31	0.258	1.83	1.43	0.209	0.20	0.78	0.721	1.73	1.42	0.189
TixSi(Es(Dis)xZo)	24	4.40	2.71	0.013	0.21	0.30	0.998	0.49	1.41	0.168	2.03	1.59	0.123	0.26	0.99	0.515	0.59	0.49	0.952
ZoxEs(Dis)xYexTi	4	1.56	0.96	0.462	0.22	0.30	0.871	0.23	0.67	0.617	1.99	1.56	0.213	0.47	1.83	0.172	0.76	0.62	0.644
Res	24	1.62			0.72			0.35			1.28			0.26			1.22		

Factors: Dis = Disturbance Category (Heavily Modified vs. Relatively Unmodified), Zo = Zone (Inner vs. Outer), Ti = Time of Sampling, Ye = Year, Es = Estuary, Si = Site. Bold values correspond to significant values for higher-level factors or interactions between non-random factors.

### Marine Trophic Index and Ecological Characteristics

Within estuary variation in the fish assemblage was not reflected by changes in the Marine Trophic Index (MTI). The MTI was relatively consistent across sampling categories and did not differ significantly by zone, estuary, year of sampling, or disturbance category (p>0.05).

In contrast, some variation was displayed in the relative abundance of estuary usage guilds. Estuarine opportunists accounted for the majority of the dataset, with 23 species comprising 82% of the fish assemblage. Estuarine Opportunists were significantly more abundant in the inner zone of all estuaries (MS_1_ = 79.92, p = 0.002) except Botany Bay. They also trended towards higher abundance in the relatively unmodified estuaries, though this was not significant (MS_1_ = 0.71, p = 0.084). Estuarine opportunists were also significantly more abundant in the first year (MS_1_ = 25.244, p = 0.016). 12 estuarine species accounted for ∼17% of the fish assemblage. No significant differences were found in the abundance of estuarine species (p>0.05). All 17 species of marine stragglers were comparatively rare, and accounted for only 1.2% of the dataset. Marine stragglers only differed significantly by estuary (MS_4_ = 1.58, p = 0.02). This was due to increased abundance of this guild in Botany Bay and the Clyde compared to other estuaries, and was primarily driven by increased abundance of *T. bailloni* in Botany Bay and *L. platycephala* in the Clyde River.

### Covariate Analyses

**Figure 6:**
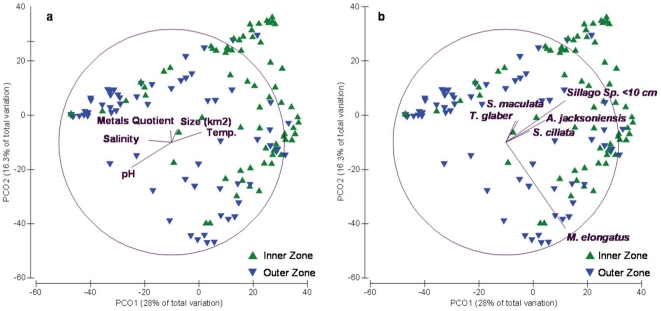
Principal Coordinated Ordination (PCO) of correlations between covariate factors and two dimensional plots of community composition by zone. a) Overlaid with physico-chemical and sediment metal vectors. b) Overlaid with vectors of top six species contributing to differences between zones. (Multiple Correlation >0.2).

Salinity, pH, and temperature were all found to have a significant relationship with the fish community composition when analyzed using a DistLM analysis (Table 7a, Figure 6a). As stated earlier, multivariate analysis of community composition found that inner and outer zones differed significantly. PCO plots indicate that the major cluster of outer zone sites was strongly related to both increased pH and salinity. In contrast, the major cluster of inner zone sites corresponded strongly to increased temperature. Sediment metals and estuary size did not correlate with the separation of zones (Figure 6a).

**Table 7:** Results of DistLM covariate analysis for a) Physico-chemical covariates and sediment metals in the full model and d) Physico-chemical covariates, sediment metals, and sediment PAH under a reduced model (inner zone only).

	a) DISTLM Covariate Analysis - Full Model				b) DISTLM Covariate Analysis - Inner Zone			
Variable	SS	F	p-value	Prop.	SS	F	p-value	Prop.
Temperature	17511.00	7.26	**0.001**	0.049	5936.60	2.59	**0.008**	0.036
Salinity	8929.80	3.61	**0.004**	0.025	4673.50	2.02	**0.045**	0.028
pH	20679.00	8.65	**0.001**	0.057	6975.00	3.07	**0.003**	0.042
Turbidity	2094.60	0.83	0.642	0.006	1543.80	0.66	0.761	0.009
Metals Quotient	3440.80	1.37	0.176	0.010	6283.10	2.75	**0.006**	0.038
Estuary Size	2504.60	0.99	0.426	0.007	4368.80	1.89	0.053	0.026
PAH Quotient	NA	NA	NA	NA	3498.80	1.50	0.125	0.021

Figure 6b plots the species which were highly correlated with the major clusters of inner and outer zone sites (those with a correlation factor >0.2). In order of the decreasing strength of this relationship these were: *M. elongatus*, *Sillago* sp. (<10 cm), *A. jacksoniensis*, *S. ciliata*, *S. maculata*, and *T. glaber*. These species were all more abundant in the inner zone (Figure 5, Figure 6b). The distributions of these species (with the exception of *M. elongatus*) approximate the vector lines of the temperature, salinity, and pH covariates. This suggests that there is a strong association between the distributions of *Sillago* sp. (<10 cm), *A. jacksoniensis*, *S. ciliata*, *S. maculata*, and *T. glaber* and temperature, salinity, and pH.

**Figure 7:**
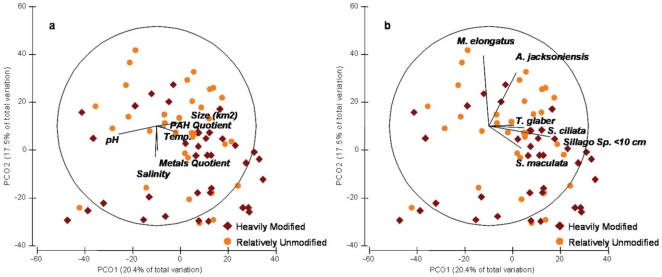
Principal Coordinated Ordination (PCO) of correlations between covariate factors and two dimensional plots of community composition by disturbance category. Data from inner zone sites only. a) Physico-chemical, sediment metals, and sediment PAH covariates. b) Plots of six highest correlating species (Multiple Correlation >0.2). Community composition does not differ significantly by disturbance category but is presented for graphical purposes (p = 0.437).

A second covariate analysis was undertaken considering inner zone data only. This allowed the inclusion of sediment PAH data as an additional covariate (which was available only from inner zone sites). As shown in Table 7b, salinity, pH, temperature, and sediment metal quotient values significantly correlated with the fish assemblages when inner zone sites were considered separately from the outer zone data (Figure 7a). Within the inner zone sites the distributions of *M. elongatus* and *A. jacksoniensis* approximated the vector lines of the sediment metals quotient. This suggests that these species were more abundant in sites with lower sediment metals contamination and lower salinity. In contrast, *T. glaber*, *S. maculata*, *S. ciliata*, and *Sillago* sp. did not show a strong relationship to sediment metals or PAH, but approximated the vector lines of the temperature and pH covariates. This suggests that these species were more abundant in areas where temperatures were higher and pH values lower (Figure 7b). While sediment metals correlate significantly in this analysis, salinity, pH, and temperature still contribute a greater proportion of the variance (Table 7b). In addition, overall community composition of the inner zone biological data does not differ significantly by disturbance category (Table 8). There is no correlation between the biological assemblage and sediment PAH levels (Table 7b). Finally, turbidity did not correlate strongly in either covariate analysis (Table 7).

**Table 8:** Multivariate analysis of community composition under a reduced model (inner zone only).

Community Composition - Inner Zone				
Source	dF	MS	F	p-value
Dis	1	8768.40	1.70	*0.154
Ye	1	3752.10	2.78	0.093
Ti	1	2152.60	1.56	0.236
Es(Dis)	4	5168.70	1.46	0.074
DisxYe	1	2131.40	1.58	0.237
DisxTi	1	3409.80	2.46	0.098
YexTi	1	4165.40	1.93	0.182
Si(Es(Dis))	12	3546.90	2.40	0.001
YexEs(Dis)	4	1350.90	0.87	0.637
TixEs(Dis)	4	1383.40	0.79	0.683
DisxYexTi	1	1624.80	0.75	0.522
YexSi(Es(Dis))	12	1549.50	1.05	0.397
TixSi(Es(Dis))	12	1760.60	1.19	0.251
YexTixEs(Dis)	4	2160.10	1.46	0.101
Res	12	1476.40		

Factors: Dis = Disturbance Category (Heavily Modified vs. Relatively Unmodified), Ti = Time of Sampling, Ye = Year, Es = Estuary, Si = Site. Bold values correspond to significant values for higher-level factors or interactions between non-random factors.

*Indicates Monte Carlo p value.

## Discussion

High levels of anthropogenic modification and sediment contamination in the estuarine environment did not strongly influence the composition, abundance, or Shannon diversity of the beach fish assemblages. We assessed these relationships within the context of environmental variability both within and between estuaries. Variation in environmental conditions between the inner and outer estuary zones were more strongly related to fish assemblages than high concentrations of contaminants. Inner zones had greater Shannon diversity, species richness, abundance, and biomass of fish while the average weight of fish was slightly higher in the outer zones. Community composition also differed between zones and some species were strongly associated with inner zones; this association was strongly correlated to pH, salinity, and temperature (but not metals or PAH contamination, turbidity, or estuary size). None of these measures of the beach fish assemblage differed between heavily modified and relatively unmodified estuaries. This indicates that differences in the fish assemblage largely follow variation in physico-chemical conditions within the estuarine system, irrespective of anthropogenic modification, substantial contamination levels, or individual variation between estuaries.

### Physico-chemical and Contamination Variables

The differences in physico-chemical variables documented in this study are consistent with the general description and understanding of environmental conditions in south-east Australian estuaries. It is well documented that the interplay between fluvial and tidal forces in these systems creates consistent differences in physico-chemical conditions within most estuaries in the region [13]. The physico-chemical parameters that we measured do not encompass the full range of environmental conditions that are expected to differ between the two zones. Some additional variables of interest that may covary with our physico-chemical measures include: wave exposure, flow rates and grain size (expected to be higher in outer zones), phytoplankton productivity, predator/prey density, sedimentation rates, and coverage of submerged aquatic vegetation (expected to be higher in the inner zones) [41], [42]. Experimental studies would be required to determine the extent to which any or all of these variables are the direct cause of the patterns we observed.

Higher levels of sediment metals in the heavily modified estuaries is consistent with the idea that urbanization, industrial development, run-off, and other sources of anthropogenic modification increase the flow of contaminants into these estuaries [22], [30]. In addition, our findings for PAHs are consistent with previous studies in the region which have found comparable PAH contamination in both relatively unmodified and heavily modified estuaries [43]. It is well known that these contaminants are highly dispersive and found in significant quantities even in otherwise pristine systems [43].

### Relationships Between Covariates and the Fish Assemblage

All biological indicators of the beach fish assemblage (community composition, abundance, species richness, Shannon diversity and fish weight) displayed significant differences between inner and outer zones. This is consistent with previous studies, which have generally found a strong relationship between fish communities and physico-chemical variables such as salinity and turbidity [10]. While correlations between physico-chemical conditions and fish distributions are expected, it is surprising that no biological indicators were found to differ significantly by anthropogenic modification and that correlations between physico-chemical covariates were always stronger than correlations with contaminant covariates. While it is well known that correlative studies are limited in their ability to identify causal relationships, there appears to be strong evidence to support the idea that variation in the physico-chemical factors within the estuary are more closely related to differences in the beach fish assemblage than contaminant concentrations. This is despite the fact that at many sites both PAH and trace metals concentrations were found to be higher than sediment quality guideline values, above which the contaminants are expected to have biological effects [32]. When the inner zone data was analyzed separately, sediment metals did correlate significantly with the biological assemblage, however, this correlation still accounted for a much smaller proportion of the variance than the physico-chemical variables. In addition, no difference was found in community composition between the inner zones of the heavily modified and relatively unmodified estuaries. Thus, even where sediment metal contamination is severe, metals do not appear to relate to community level impacts in this post-settlement fish assemblage.

The extent to which entire fish communities or populations may be affected when contaminants have negative consequences for individuals is poorly understood [6]. The physiological mechanisms by which contaminants affect the health of individual fish have been previously investigated and a great deal of literature examines the presence, biomagnification, toxicology, and biomarker response of contaminants in marine fishes [44], [45]. Fish primarily take up contaminants through ingestion of contaminated food particles and to a lesser extent from water that passes over the gill membranes [28]. Once ingested, contaminants move through a wide variety of physiological and chemical pathways, many of which have detrimental effects for the individual. However, the extent to which these organismal effects translate into community or population level impacts is rarely studied [6]. In theory, contaminants may affect fish populations and diversity by reducing fish health and survivorship [46], by reducing growth and reproductive success [47], by reducing the abundance of prey species, and by increasing instances of deformity [8]. Ultimately any of these mechanisms could link contaminant exposure to organismal effects and ultimately population level impacts. Response mechanisms may be species specific and a lack of knowledge in this area somewhat hinders our ability to detect and understand community level impacts of contaminants.

### Ecological Characteristics and Life History Stages

The findings of this study contrast directly with our findings for larval fish communities in these same estuaries. In previous studies we have shown that early life-history stages (larval fish) varied substantially between heavily modified and relatively unmodified estuaries, while showing a strong relationship to sediment metals [4]. We have also demonstrated that in these estuaries anthropogenic stressors appear to primarily affect estuarine taxa and benthic egg layers [9]. In the current study estuarine opportunist species accounted for the majority of the dataset and were the major driver of differences in fish assemblages between the inner and outer zone of estuaries. However, none of the estuary usage guilds differed significantly by disturbance category, and there was no strong evidence to suggest differential sensitivity among estuarine taxa.

The disjuncture between the results for the larval and beach fish assemblages may be due to a variety of factors. First, it should be noted that the larval fish assemblage sampled in previous studies were more diverse than this beach fish assemblage, and that many of the larval species are closely associated with biogenic habitats such as seagrass and mangroves (unlike the beach fish). This may explain the more pronounced impacts observed for larvae [4]. Second, because the majority of beach fish species are estuarine opportunists, many of them are not captured in large numbers by larval sampling in estuaries. The larval studies indicated that the majority of anthropogenic impacts are seen among estuarine resident taxa, a group which is underrepresented in this study [9].

The only major species encountered in large numbers in both the beach seine and larvae studies were *S. ciliata*, *S. maculata*, *G. subfasciatus*, *H. vittatus*, *F. lentiginosus*, and *A. jacksoniensis*. Of these, *A. jacksoniensis* was the only species which differed significantly by modification in both studies, being more abundant in the relatively unmodified estuaries. However, the scale of difference was not equal; whereas post-settlement *A. jacksoniensis* were ∼1.5 times more abundant in the relatively unmodified estuaries, larvae were ∼14 times more abundant. This indicates that there is a greater difference in the abundance of this species between heavily modified and relatively modified estuaries at the larval stage. This is consistent with the hypothesis that fish are more sensitive at their larval stage and that impacts are more easily detected on larval assemblages. However, it also suggests that the relationship between larval and post-settlement abundance is not straightforward, and hence that impacts at the larval stage may not directly translate into impacts at the post-settlement stage. The non-linear relationship between larval and post-settlement abundance has been well documented in the supply-side ecology literature [48].

### Conclusion

The importance of chemical contamination as an ecosystem stressor will depend on site attributes and variability in environmental conditions [20]. Our study suggests that variation in physico-chemical factors has a much greater influence on the beach fish assemblage than the extent of anthropogenic modification or pollution in the estuaries examined in this study. Significant differences in physico-chemical conditions exist within these estuaries and these factors are highly associated with the distributions of some fish species and the composition of the fish community generally. Despite comparatively high levels of anthropogenic modification and contamination, there did not appear to be a large effect on the portion of the fish assemblage examined in this study. However, fish living in more sensitive biogenic habitats such as coral reefs and seagrass beds may be affected to a much greater degree by modification and contamination, as the biogenic habitat itself may be degraded by these stressors. Unlike benthic larval fish assemblages, the estuarine beach fish do not represent a sensitive indicator of contaminant impact. Other forms of environmental disturbance which have a large scale influence on physico-chemical conditions, such as diversion of freshwater flows, coastal alteration, and sea level rise, may have greater potential to affect the beach fish assemblage than the current input of marine contamination. Ultimately, conservation and management efforts which include a consideration of physico-chemical variables will be more effective at protecting these fish assemblages.

## Supporting Information

Appendix S1 **Average beach fish abundance identified to lowest taxonomic level by estuary and zone.** Heavily modified estuaries – Port Jackson, Botany Bay and Port Kembla. Relatively unmodified estuaries – Port Hacking, Jervis Bay and the Clyde River. Abbreviations - Life Cycle Guild: EO = Estuarine Opportunist, E = Estuarine, MS = Marine Straggler. Trophic level values taken from [33].
(DOC)

## References

[1] Lotze HK, Lenihan HS, Bourque BJ, Bradbury RH, Cooke RG (2006). Depletion, degradation, and recovery potential of estuaries and coastal seas.. Science.

[2] Kennish MJ (2002). Environmental threats and environmental future of estuaries.. Environmental Conservation.

[3] Beck MW, Heck KL, Able KW, Childers DL, Eggleston DB (2001). The identification, conservation, and management of estuarine and marine nurseries for fish and invertebrates.. Bio Science.

[4] McKinley AC, Miskiewicz A, Taylor MD, Johnston EL (2011). Strong links between metal contamination, habitat modification and estuarine larval fish distributions.. Environmental Pollution.

[5] Johnston E, Roberts DA (2009). Contaminants reduce the richness and evenness of marine communities: A review and meta-analysis.. Environmental Pollution.

[6] McKinley A, Johnston EL (2010). Impacts of contaminant sources on marine fish abundance and species richness: A review and meta-analysis of evidence from the field.. Marine Ecology Progress Series.

[7] Miskiewicz AG, Gibbs PJ (1994). Organochlorine pesticides and hexachlorobenzene in tissues of fish and invertebrates caught near a sewage outfall.. Environmental Pollution.

[8] Kingsford MJ, Suthers IM, Gray CA (1997). Exposure to sewage plumes and the incidence of deformities in larval fishes.. Marine Pollution Bulletin.

[9] McKinley AC, Foster-Thorpe C, Miskiewicz A, Taylor MD, Johnston EL (in review). Anthropogenic activities differentially impact fish guilds: The importance of understanding life history characteristics.. Journal of Applied Ecology.

[10] Potter IC, Hyndes GA (1999). Characteristics of the ichthyofaunas of southwestern Australian estuaries, including comparisons with holarctic estuaries and estuaries elsewhere in temperate Australia: A review.. Australian Journal of Ecology.

[11] Rakocinski CF, Baltz DM, Fleeger JW (1992). Correspondence between environmental gradients and the community structure of marsh-edge fishes in a Louisiana estuary.. Marine Ecology Progress Series.

[12] Taylor MD, Laffan SD, Fielder DS, Suthers IM (2006). Key habitat and home range of mulloway Argyrosomus japonicus in a south-east Australian estuary: finding the estuarine niche to optimise stocking.. Marine Ecology Progress Series.

[13] Roy P, Williams R (2001). Structure and function of south-east Australian estuaries.. Estuarine, Coastal and Shelf Science.

[14] Masselink G, Short AD (1993). The effect of tide range on beach morphodynamics and morphology: A conceptual beach model.. Journal of Coastal Research.

[15] Castilla JC (1983). Environmental impact in sandy beaches of copper mine tailings at Chañaral, Chile.. Marine Pollution Bulletin.

[16] Rice C (2006). Effects of shoreline modification on a Northern Puget Sound beach: Microclimate and embryo mortality in surt smelt (Hypomesus pretiosus).. Estuaries and Coasts.

[17] Edgar GJ, Shaw C (1995). The production and tropic ecology of shallow-water fish assemblages in southern Australia. III. General relationships between sediments, seagrasses, invertebrates and fishes.. Journal of Experimental Marine Biology and Ecology.

[18] Deegan L (2002). Lessons learned: The effects of nutrient enrichment on the support of nekton by seagrass and salt marsh ecosystems.. Estuaries and Coasts.

[19] Reopanichkul P, Schlacher TA, Carter RW, Worachananant S (2009). Sewage impacts coral reefs at multiple levels of ecological organization.. Marine Pollution Bulletin.

[20] Burton GA, Johnston EL (2010). Assessing contaminated sediments in the context of multiple stressors.. Environmental Toxicology and Chemistry.

[21] Underwood AJ (1994). On beyond BACI: Sampling designs that might reliably detect environmental disturbances.. Ecological Applications.

[22] Scanes P (2010). NSW Estuarine catchment disturbance ranks.

[23] NSWDNR (2010). Estuaries in New South Wales.. http://www.naturalresources.nsw.gov.au/estuaries/inventory/index_ns.shtml.

[24] ANRA (2009). Land use - Clyde River - Jervis basin.. http://www.anra.gov.au/topics/land/landuse/nsw/basin-clyde-river.html.

[25] DSEWPC (2011). Interim biogeographic regionalization of Australia.. http://www.environment.gov.au/parks/nrs/science/bioregion-framework/ibra/index.html.

[26] Gomon M, Bray D, Kuiter R (2008). Fishes of Australia's southern coast.

[27] USEPA (2007). Method 3051A microwave assisted acid digestion of sediments, sludges and oils. Environmental Protection Agency.

[28] Dallinger R, Prosi F, Segner H, Back H (1987). Contaminated food and uptake of heavy metals by fish: a review and a proposal for further research.. Oecologia.

[29] Hall BD, Bodaly RA, Fudge RJP, Rudd JWM, Rosenberg DM (1997). Food as the Dominant Pathway of Methylmercury Uptake by Fish.. Water, Air, & Soil Pollution.

[30] Knott NA, Aulbury J, Brown T, Johnston EL (2009). Contemporary ecological threats from historical pollution sources: impacts of large-scale resuspension of contaminated sediments on sessile invertebrate recruitment.. Journal of Applied Ecology.

[31] Long ER (2006). Calculation and uses of mean sediment quality guideline quotients: A critical review.. Environmental Science & Technology.

[32] ANZECC (2000). Australian and New Zealand guidelines for fresh and marine water quality.

[33] Froese R, Pauly D (2010). Fishbase.. http://www.fishbase.org.

[34] Pauly D, Watson R (2005). Background and interpretation of the ‘Marine Trophic Index’ as a measure of biodiversity.. Philosophical Transactions of the Royal Society BIology.

[35] Elliott M, Whitfield AK, Potter IC, Blaber SJM, Cyrus DP (2007). The guild approach to categorizing estuarine fish assemblages: a global review.. Fish and Fisheries.

[36] Neira FJ, Miskiewicz AG, Trnski T (1998). Larvae of temperate Australian fishes: A laboratory guide for larval fish identification.

[37] Anderson MJ (2001). A new method for non-parametric multivariate analysis of variance.. Austral Ecology.

[38] McArdle BH, Anderson MJ (2001). Fitting multivariate models to community data: a comment on distance-based redundancy analysis. Ecology.

[39] Anderson MJ (2003). PCO - Principal Coordinate Analysis: A computer program.

[40] Quinn G, Keough M (2002). Experimental design and data analysis for biologists.

[41] Iverson RL (1990). Control of marine fish production.. Limnology and Oceanography.

[42] Clark BM (1997). Variation in surf-zone fish community structure across a wave-exposure gradient.. Estuarine, Coastal and Shelf Science.

[43] Maher WA, Aislabie J (1992). Polycyclic aromatic hydrocarbons in nearshore marine sediments of Australia.. Science of the Total Environment.

[44] Costello, Mark J, Read, Paul (1994). Toxicity of sewage sludge to marine organisms: A review.. Marine Environmental Research.

[45] van der Oost R, Beyer J, Vermeulen NPE (2003). Fish bioaccumulation and biomarkers in environmental risk assessment: a review.. Environmental Toxicology and Pharmacology.

[46] Robinet TT, Feunteun EE (2002). Sublethal effects of exposure to chemical compounds: A cause for the decline in Atlantic eels?. Ecotoxicology.

[47] Waring CP, Stagg RM, Fretwell K, McLay HA, Costello MJ (1996). The impact of sewage sludge exposure on the reproduction of the sand goby, Pomatoschistus minutus.. Environmental Pollution.

[48] Roughgarden J, Gaines S, Possingham H (1988). Recruitment dynamics in complex life cycles.. Science.

