# Supplementary material for: High Levels of Sediment Contamination Have Little Influence on Estuarine Beach Fish Communities
Source: PLoS One. 2011 Oct 19;6(10):e26353. doi: 10.1371/journal.pone.0026353 (PMC3198393; doi:10.1371/journal.pone.0026353)
Supplement: Appendix S1 — Average beach fish abundance identified to lowest taxonomic level by estuary and zone. Heavily modified estuaries – Port Jackson, Botany Bay and Port Kembla. Relatively unmodified estuaries – Port Hacking, Jervis Bay and the Clyde River. Abbreviations - Life Cycle Guild: EO = Estuarine Opportunist, E = Estuarine, MS = Marine Straggler. Trophic level values taken from [33]. (DOC) [file pone.0026353.s001.doc]

**Appendix S1 - Average beach fish abundance identified to lowest taxonomic level by estuary and zone. Heavily modified estuaries – Port Jackson, Botany Bay and Port Kembla. Relatively unmodified estuaries – Port Hacking, Jervis Bay and the Clyde River. Abbreviations - Life Cycle Guild: EO = Estuarine Opportunist, E = Estuarine, MS = Marine Straggler. Trophic level values taken from [33].**
